# Rituximab in Myasthenia Gravis: a real-world study using inverse probability of treatment weighting

**DOI:** 10.3389/fneur.2026.1882884

**Published:** 2026-07-02

**Authors:** Yong Lin Wang, Chao Zhu, Mahima Kapoor, Gary Cutter, Carolina Barnett-Tapia, Helmut Butzkueven, Wenwen Zhang, Gabor Lovas, Csilla Rozsa, Jeannine Heckmann, Stefan Blum, Laurie McLaughlin, Katherine Buzzard, Yi Chao Foong, Elisabeth Chroni, Dimitra Veltsista, Belinda Cruse, Mina Botrous, Stephen Reddel, Mastura Monif, Anneke van der Walt

**Affiliations:** 1 Department of Neuroscience, School of Translational Medicine, Monash University, Melbourne, VIC, Australia; 2 Department of Neurology, Alfred Health, Melbourne, VIC, Australia; 3 Department of Biostatistics, University of Alabama in Birmingham, Birmingham, AL, United States; 4 Division of Neurology, Department of Medicine, University Health Network, University of Toronto, Toronto, ON, Canada; 5 Department of Neurology, Jahn Ferenc Hospital, Budapest, Hungary; 6 Neurology Research Group, Neuroscience Institute, University of Cape Town, Cape Town, South Africa; 7 Department of Neurology, Princess Alexandra Hospital, Wooloongabba, QLD, Australia; 8 Department of Neurosciences, Box Hill Hospital, Box Hill, VIC, Australia; 9 Eastern Health Clinical School, Monash University, Box Hill, VIC, Australia; 10 Depatment of Neurology, Royal Hobart Hospital, Tasmanian Health Service, Hobart, TAS, Australia; 11 MS Research Flagship, Menzies Institute of Medical Research, University of Tasmania, Hobart, TAS, Australia; 12 Neuromuscular Unit, Department of Neurology, University of Patras, Patras, Greece; 13 Department of Neurology, Royal Melbourne Hospital, Melbourne, VIC, Australia; 14 Faculty of Medicine, Health and Dentistry, Department of Medicine (RMH), The University of Melbourne, Melbourne, VIC, Australia; 15 Brain and Mind Centre, Camperdown, NSW, Australia

**Keywords:** immunosuppressive agents, Myasthenia Gravis, Propensity Score, real world evidence, rituximab

## Abstract

**Background:**

The role of rituximab in the treatment of myasthenia gravis (MG) remains uncertain due to limited randomized controlled evidence and heterogeneous observational data. While rituximab is often used in refractory MG, its comparative effectiveness against other non-steroidal immunosuppressive therapies (NSISTs) has yet to be fully clarified.

**Objective:**

To evaluate the effectiveness of rituximab compared to a second NSIST in achieving a composite clinical outcome in acetylcholine-receptor-antibody (AChR-Ab) positive MG patients using causal inference methods to adjust for confounding.

**Methods:**

We conducted a retrospective cohort study of AChR-Ab positive MG patients treated with rituximab or a second NSIST. The primary outcome was time to achieving a composite clinical endpoint representing a patient acceptable symptom state (PASS): Myasthenia Gravis Composite (MGC) score ≤ 3, daily corticosteroid dose ≤ 5 mg prednisolone equivalent, and no rescue therapy use in the preceding month. To reduce confounding by indication and improve causal comparability between treatment groups, inverse probability of treatment weighting (IPTW) based on propensity scores was used to balance baseline covariates. Cox proportional hazards models were applied to estimate the effect of treatment on time to outcome.

**Results:**

169 patients entered into the time-to-event analysis, and after IPTW adjustment, baseline characteristics between treatment groups were balanced. There was no statistical difference in the hazard ratio between Rituximab compared to a second NSIST in achieving the composite clinical outcome (HR = 1.27, 95% CI 0.66–2.45, *p* = 0.48).

**Conclusion:**

In this IPTW-adjusted analysis, rituximab did not improve the time-to-improvement compared to a second NSIST in AChR-Ab-positive MG.

## Introduction

Myasthenia gravis (MG) is a rare autoimmune disease characterised by fluctuating and fatigable weakness due to antibody-mediated dysfunction at the neuromuscular junction. Anti-acetylcholine receptor (AChR) antibody is the most common autoantibody found in MG, with a direct role in pathogenesis (1).

Most MG patients with generalised disease will initially require treatment with corticosteroids, which, in the majority of patients, reliably induces remission of symptoms (2). Intravenous immunoglobulins (IVIG) or plasma exchange (PLEX) may also be given in severe disease manifestations (3), which can be life-saving in myasthenic crises. The withdrawal of corticosteroids is a crucial clinical outcome, given their highly unfavourable profile of adverse effects, which have significant implications for quality of life.

Return of symptoms with weaning of corticosteroid therapy is generally an indication for additional steroid-sparing immunosuppressive therapy. Currently, the antiproliferative agents azathioprine and mycophenolate remain the first-line non-steroidal immunosuppressant therapies (NSIST) used in AChR MG, despite limited randomised controlled trial evidence. Both drugs have a lengthy latency to effect of greater than 6 to 12 months (4, 5). Other lines of immunosuppression also include the calcineurin inhibitors cyclosporine (6) and tacrolimus (7), as well as methotrexate (8) and cyclophosphamide (9), some of which are accompanied by greater toxicities. Rituximab is an anti-CD20 monoclonal antibody whose successful use in MG was first published in a case report of refractory disease in 2001 (10). B-cell depletion therapies have a theoretically plausible mechanism of action in an antibody-mediated disease, but evidence of efficacy remains equivocal. In addition, the duration of clinical response after treatment is not necessarily correlated to B-cell repopulation due to several factors, and re-treatment intervals for rituximab are not clearly defined. Several observational studies and systematic reviews generally support its use in MG, mainly in the setting of treatment-refractory MG (11, 12). A meta-analysis performed on 15 small case series found an overall response rate of 83.9% to rituximab as measured by improvement in Myasthenia Gravis Foundation of America Post-Intervention Status (MGFA-PIS) (13). Two randomized clinical trials have also offered differing conclusions, where rituximab use in early MG disease improved clinical outcomes (14), while rituximab used as an add-on in severe disease did not result in steroid-sparing effect (15). Despite clinical equipoise, in the current MG landscape, rituximab remains an important immunomodulatory therapy.

In this study, we use real-world data from the MGBase registry to evaluate the relative efficacy of rituximab compared to NSISTs in AChR MG (16).

## Methods

This is a retrospective cohort study of patients enrolled in the MGBase Registry between its launch in October, 2021 and April, 2025. MGBase is the first international observational registry for MG, launched in 2021 (16). Ethics approval for the MGBase registry was granted by local ethics committees in participating centres. Written or verbal consent has been obtained from all enrolled patients. The MGBase minimum dataset requires capture of birthdate, gender, diagnosis, onset date at registration, followed by at a minimum annual assessments of modified Rankin Score and Myasthenia Gravis Composite (MGC) score. Key data, including treatments, clinical scores and diagnostic tests, are collected during clinical encounters. Treatments alongside their dosage and frequency are recorded on commencement, cessation as well as change of dosage.

### Study population

Inclusion required patients with AChR-Ab positive serology status who had exposure to either rituximab (the rituximab cohort) or a non-steroidal immunosuppressant (the NSIST cohort) defined as any of mycophenolate, azathioprine, cyclosporine, tacrolimus, or methotrexate. Patients were required to undergo a clinical assessment with the Myasthenia Gravis Composite (MGC) score before commencing their treatment (either rituximab or the second NSIST), and at least two subsequent assessments with MGC scores to determine clinical stability. Given the real-world nature of MGBase, rituximab administration patterns varied. Exposure was defined as the administration of at least one dose of rituximab. We extracted data on the specific dosing patterns, specifically noting induction (e.g., two 1,000 mg doses a fortnight apart or weekly 375 mg/m^2^ for four weeks) versus ongoing maintenance treatment (over years). For the primary analysis, all rituximab exposures were analysed as a single cohort to maximise statistical power. Furthermore, patients in the NSIST cohort were required to be on their second NSIST to match clinical practice with rituximab use, where overwhelmingly rituximab is not used as a first-line therapy. Thus, the patients in the NSIST cohort had already been exposed to a different NSIST from the list above.

### Study end points

The primary endpoint was the achievement of a composite outcome defined as a Patient Acceptable Symptom State (equivalent to an MGC score of less than or equal to 3) (17), a daily corticosteroid dose of less than or equal to 5 mg prednisolone-equivalent, and no requirement for rescue PLEX or IVIG within the preceding 30 days. This outcome is similar to the level 1 outcome in the MG Status and Treatment Intensity (MGSTI) index, noting that MGBase uses MGC as a primary and minimum outcome, but not MGFA post-intervention status (PIS), which was used in the MGSTI (18). We also analysed a secondary endpoint defined by the reduction of daily corticosteroid dose to less than or equal to 5 mg prednisolone-equivalent, without recent requirement for rescue therapies, to assess the effect of the treatments on reducing corticosteroid dosage alone. To account for the persistence of benefit, we required achievement of these composite outcomes on two consecutive clinical visits. Baseline was defined at the most recent visit prior to the commencement of the study therapy (that is, rituximab or the second NSIST). Follow-up occurred until the achievement of the primary end point or until the last visit recorded in MGBase.

### Statistical analysis

A time-to-event analysis was conducted to assess the effect of rituximab treatment on achieving the composite clinical outcome in patients, compared with treatment with a second NSIST. Given the potential for confounding due to baseline characteristic imbalance, inverse probability of treatment weighting (IPTW) was applied using covariate balancing propensity scores to balance the treatment cohorts. IPTW is a causal inference approach that reduces confounding by weighting participants to create pseudo-populations that are comparable across observed covariates, conceptually mimicking a randomised control trial (19). The core assumption of exchangeability requires no unmeasured confounding factors. Covariate balance was implemented using the *weightit* package in *R* (20). The balancing targeted covariates, including age, gender, number of previous ISTs, baseline (pre-treatment) prednisolone dose, baseline (pre-treatment) MGC score and thymectomy status, and a standardised mean difference (SMD) of below 0.1 for all covariates was required to indicate good balance. A Cox proportional hazards model was then fitted using the *survival* package in R (21). The time-to-event outcome was modelled with treatment (rituximab vs. second NSIST) as the primary predictor. Weights from IPTW were incorporated, with robust (sandwich) standard errors calculated to account for uncertainty due to the use of estimated weights, improving the reliability of inference. In addition, Kaplan–Meier survival curves were plotted to visually compare the time to achieving the outcome between the two cohorts. A log-rank test was performed to assess the difference in survival curves.

The global Schoenfeld test was performed to confirm the proportional hazards assumption held for the model. All statistical tests were 2-sided, with a statistical significance defined as *p* ≤ 0.05. Statistical analysis was performed with *R* (22) using *survival* (21) for survival analysis and *ggplot2* (23) for plot generation.

### Sensitivity analysis

The robustness of the primary outcome was tested with several sensitivity analyses. IPTW balanced analysis was repeated with adjustments to the definition of the composite outcome. Given the risk of prednisolone use being an under-captured attribute in the registry, the composite outcome was adjusted by omitting the criterion for daily prednisolone dose of 5 mg or less. Analysis was also repeated where attainment of the composite outcome was required only on one visit (compared to two consecutive visits), given the possibility of significant time-gaps between visits entered into a real-world registry. To address the inherent treatment heterogeneity associated with real-world rituximab use, a subgroup analysis was performed to evaluate the impact of treatment intensity. The rituximab cohort was stratified into a single induction course subgroup (treat course < 30 days in duration) and a maintenance treatment subgroup (treatment course > 180 days). Pairwise IPTW-balanced comparisons were made between each subgroup against a second NSIST. Analysis was also undertaken with inclusion of seronegative patients, and with exclusion of patients with thymectomy occurring in the preceding two years. A multivariate Cox proportional hazards model was fitted to the study cohort without IPTW balancing, adjusted for rituximab treatment, gender, age, thymectomy status, number of previous ISTs, baseline MGC and prednisolone dosage.

## Results

**Figure 1:**
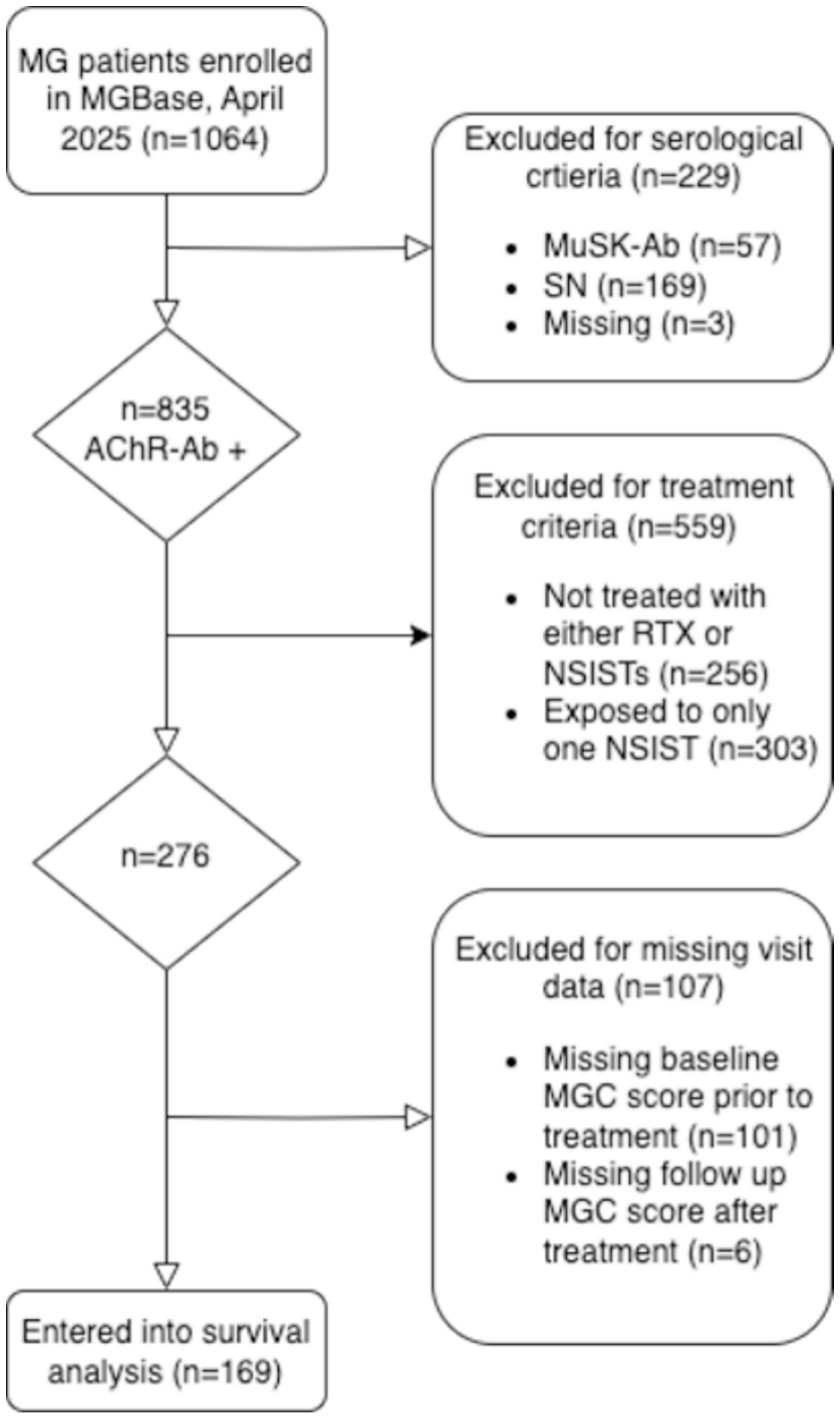
Participant flow diagram. MuSK, muscle-specific kinase; SN, seronegative; RTX, rituximab; NSIST, non-steroidal immunosuppressant therapy; MGC, Myasthenia Gravis composite score.

Of the 1,064 patients with MG enrolled at the time of the April 2025 MGBase data extract, 169 met the treatment and follow up requirements of the time-to-event analysis (Figure 1).

Demographic and clinical features of the overall MGBase cohort and the cohort who met criteria for the planned time-to-event analysis are summarized in Table 1. In the 169 included patients, follow-up duration was on average longer than the overall cohort (*n* = 1,064), and over their lifetime, they were exposed to a larger number of NSIST. Age, sex and thymectomy status were comparable between the two cohorts. The study cohort did have higher worst-MGC scores recorded over the observation period.

**Table 1:** Characteristics of the patients in both the overall MGBase cohort and the study subgroup. For cohort comparisons, a Chi-squared test is used to compare categorical variables, a t-test for continuous variables, and a Kruskal-Wallis test for non-normal continuous variables.

Characteristic	Subcategory	Overall MGBase cohort	Study cohort	*p*
Total *n*		1,064	169	
Sex (%)	Male	490 (46.1)	68 (40.2)	0.18
	Female	574 (53.9)	101 (59.8)	
Age at last visit, mean years (SD)		58.08 (18.28)	56.13 (17.88)	0.20
Age at disease onset, mean years (SD)		48.21 (20.31)	45.29 (20.52)	0.083
Follow up duration, median years [IQR]		1.67 [0.12, 5.00]	4.93 [2.00, 7.99]	<0.001
Onset (%)	Generalised	653 (61.4)	119 (70.4)	0.03
	Ocular	411 (38.6)	50 (29.6)	
Serology (%)	Seronegative	169 (15.9)	0 (0.0)	<0.001
	AChR-Ab	835 (78.7)	169 (100.0)	
	MuSK-Ab	57 (5.4)	0 (0.0)	
Thymectomy (%)	None	739 (69.5)	114 (67.5)	0.49
	Non-thymoma	224 (21.1)	34 (20.1)	
	Thymoma	101 (9.5)	21 (12.4)	
Lifetime Number of ISTs, mean (SD)		1.46 (1.22)	2.82 (1.03)	<0.001
Worst MGC score, mean (SD)		10.10 (8.73)	11.59 (8.66)	0.041

Table 2 describes the 169 patients of the study cohort before weighting, where more patients were exposed to rituximab compared to NSIST exposure alone. The cohorts were similar with respect to age, sex, and baseline prednisolone dose. While the NSIST cohort was selected only on the second NSIST commencement (therefore, by definition, had exposure to 1 previous NSIST), the rituximab cohort had, on average, been exposed to 1.19 previous NSISTs. Notably, there are differences in usage patterns between rituximab and the standard NSISTs, as reflected in the treatment duration for each cohort. The median duration of treatment for rituximab was 385 days (1.05 years), with a large proportion of the cohort (33 of 117 patients, 28%) having had only one dose or one induction treatment (two doses over two weeks), while standard NSIST treatment was more enduring, with a median treatment duration of 598 days. The baseline MGC score prior to treatment commencement between the groups differed by approximately 4 points (NSIST 6.65; rituximab 10.69, *p* = 0.004). Compared to the NSIST cohort, the rituximab cohort had a larger proportion of post-thymectomy patients, which did not reach statistical significance (37.6% vs. 21.2%, *p* = 0.37). The choice of second NSIST initiated was predominantly methotrexate (*n* = 21) and mycophenolate (*n* = 18), with azathioprine (*n* = 5), tacrolimus (*n* = 5) and cyclosporin (*n* = 3) contributing smaller proportions. During the study period, 27 patients in the rituximab cohort were subsequently commenced on an NSIST, while 9 patients in the NSIST cohort subsequently commenced on another NSIST. After IPTW balancing, the weighted pseudo-population included approximately 175 patients per cohort. However, due to variability in the weights, the effective sample size was reduced to 39.0 (NSIST cohort) and 106.3 (rituximab cohort), which reflects the precision available for statistical inference from which robust standard errors and all *p-*values are calculated. All covariates were balanced between the rituximab and the NSIST cohorts, with an absolute difference in means < 0.1 across all covariates.

**Table 2:** Characteristics of the patients in the time-to-event analysis, stratified by treatment, before and after IPTW weighting.

Characteristic	Subcategory	Unadjusted				IPTW adjusted pseudo-cohort		
		IST	RTX	*p*	Standardised mean difference	IST	RTX	Standardised mean difference
Cohort size		52	117			175	175	
Gender (%)	*M*	32 (61.5)	69 (59.0)	0.89	0.052	71.5 (40.9)	71.5 (40.9)	<0.001
	*F*	20 (38.5)	48 (41.0)			103.5 (59.1)	103.5 (59.1)	
Age at treatment, mean years (SD)		51.81 (18.67)	52.77 (17.64)	0.75	0.053	51.89 (18.01)	51.89 (18.24)	<0.001
Treatment duration, median days [IQR]		598.50 [172.50, 1986.25]	385.00 [15.00, 931.00]	0.003 (non-normal)				-
Thymectomy (%)	None	41 (78.8)	73 (62.4)	0.11	0.37	120.1 (68.6)	120.1 (68.6)	<0.001
	Non-thym	7 (13.5)	27 (23.1)			35.4 (20.2)	35.4 (20.2)	
	Thymoma	4 (7.7)	17 (14.5)			19.5 (11.1)	19.5 (11.1)	
Baseline MGC score, mean (SD)		6.65 (5.53)	10.69 (9.15)	0.004	0.53	8.85 (6.50)	8.85 (8.55)	<0.001
Baseline daily prednisolone dose, mean mg (SD)		13.10 (16.77)	14.21 (24.07)	0.76	0.054	14.62 (17.31)	14.63 (24.21)	<0.001
Number of ISTs before treatment, mean (SD)		1.00 (0.00)	1.19 (0.88)	0.13	0.30	1.00 (0.00)	1.00 (0.87)	<0.001

**Figure 2:**
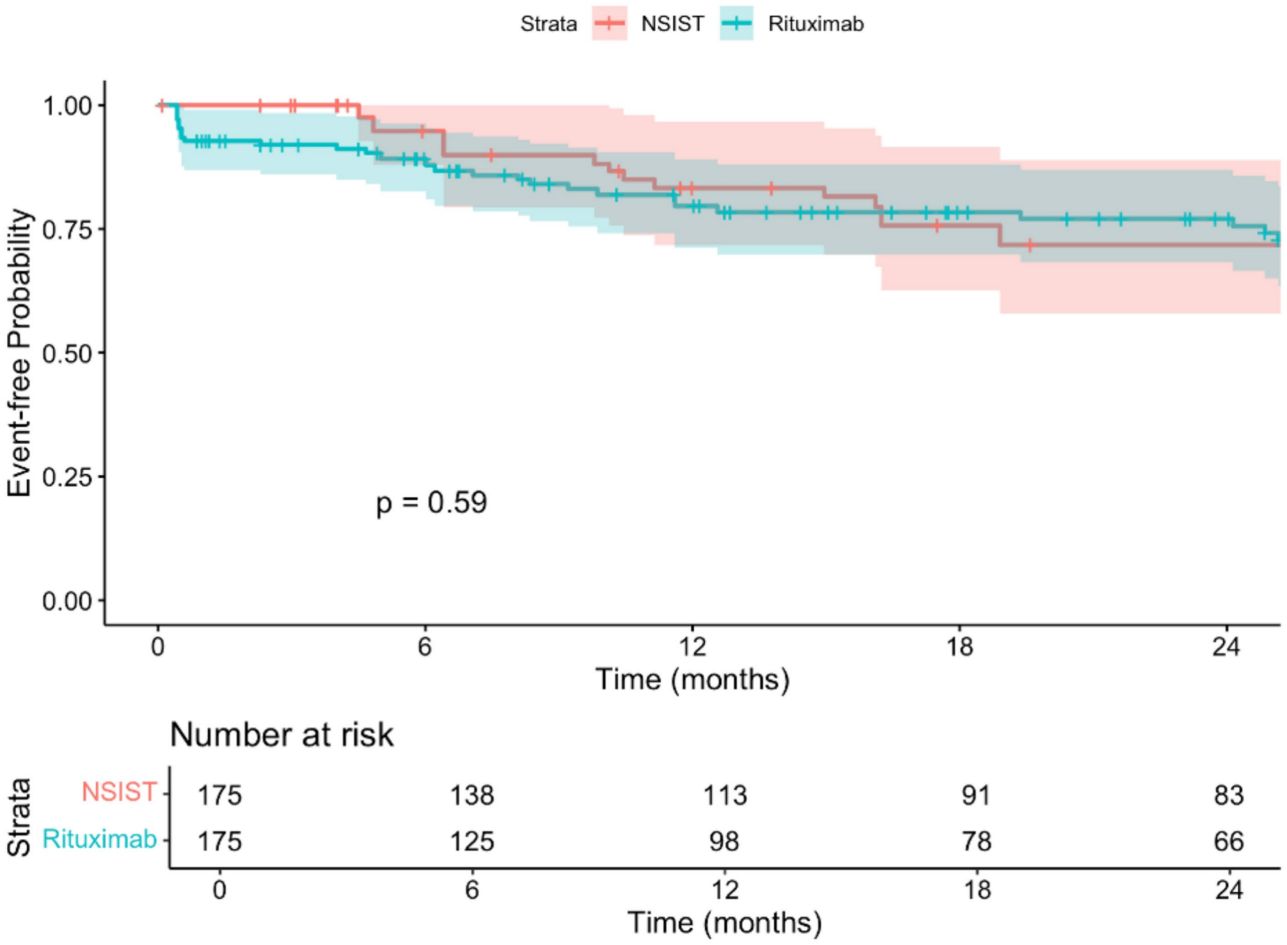
Kaplan–Meier curve - achievement of primary composite treatment outcome (MGC ≤ 3, daily steroid dosage of ≤ 5 mg prednisolone equivalent, no rescue therapy with IVIG or PLEX within 4 weeks) stratified by treatment with Rituximab or NSIST.

The composite outcome was reached by 53 of 169 patients, 32 in the rituximab cohort and 21 in the NSIST cohort, over a median follow-up duration of 4.9 years. Kaplan–Meier curves for the two cohorts are shown in Figure 2, where the log-rank test did not find a significant difference (*p* = 0.59). The univariate Cox Proportional Hazards model (Table 3) also demonstrated no significant difference between cohorts (HR = 1.27, 95% CI 0.66–2.45, *p* = 0.48). The proportional hazards assumption was tested using scaled Schoenfeld residuals and no violations were observed. Weight diagnostics showed adequate overlap and no extreme weight values. The effective sample sizes and covariate balance were satisfactory, confirming validity of the pseudo-population for causal inference.

**Table 3:** Cox proportional hazards analysis for achievement of primary composite treatment outcome (MGC ≤ 3, daily steroid dosage of ≤ 5 mg prednisolone equivalent, no rescue therapy with IVIG or PLEX within 4 weeks) of rituximab compared to second NSIST.

Group	Patients	Effective sample size (IPTW)	Events	b*	HR	95% CI	*SE*	Robust SE	*z*	*p*
NSIST	52	39.0	21		1.00 (ref)					
Rituximab	117	106.3	32	0.24	1.27	[0.66, 2.45]	0.20	0.34	0.71	0.48

**Figure 3:**
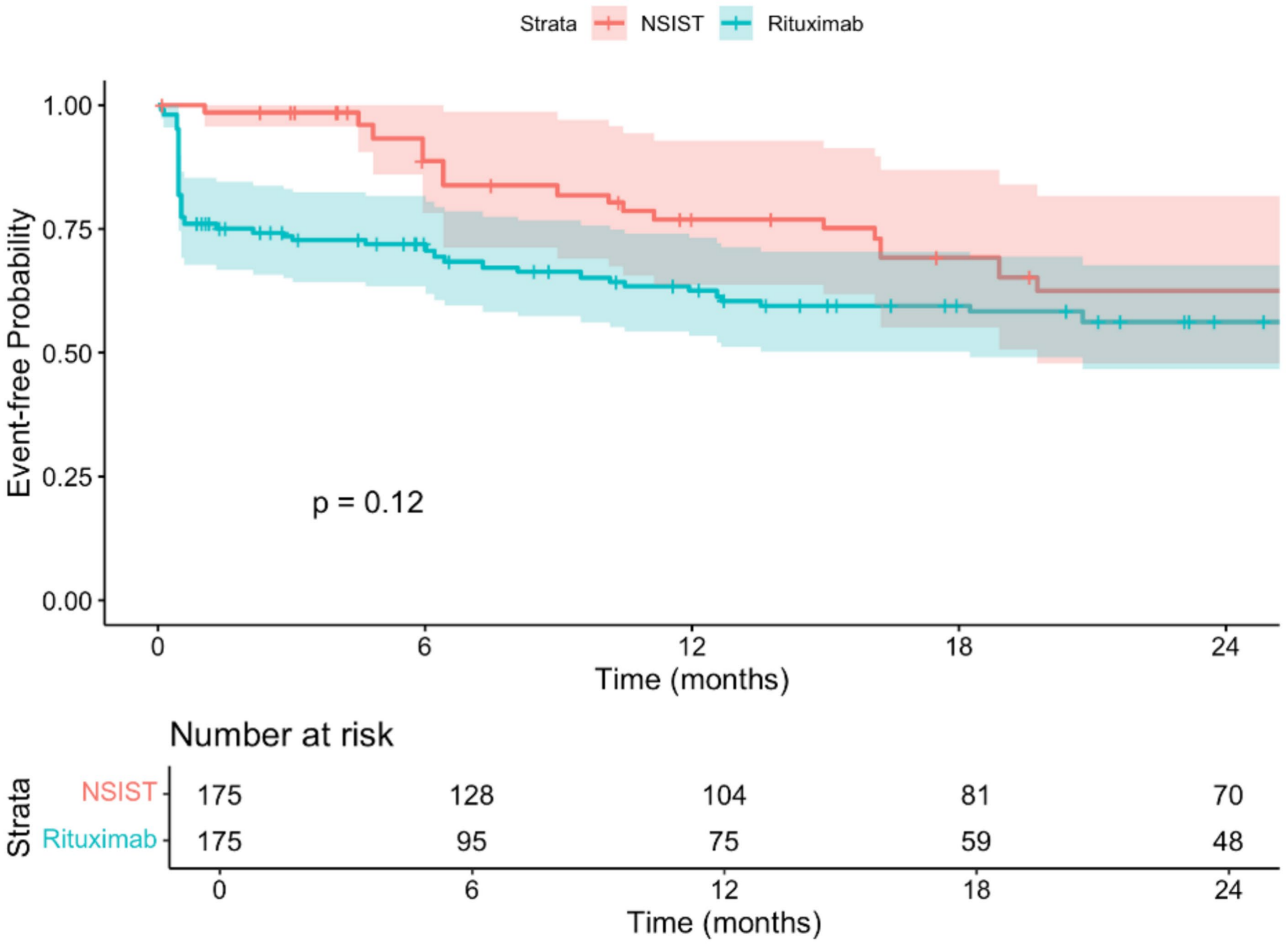
Kaplan–Meier curve - achievement of secondary composite treatment outcome (daily steroid dosage of ≤ 5 mg prednisolone equivalent and no rescue therapy with IVIG or PLEX within 4 weeks) stratified by treatment with Rituximab or NSIST.

We also analysed a secondary outcome that required achievement of a corticosteroid dose of less than or equal to 5 mg daily prednisolone-equivalent and the absence of rescue therapy with IVIG or PLEX in the preceding month (Table 4). This also demonstrated no difference between groups (HR = 1.59, 95% CI 0.95–2.67, *p* = 0.08). Similar results were obtained on Kaplan–Meier plots with non-significant log-rank testing (*p* = 0.12) (Figure 3). In this analysis, 83 patients achieved the composite outcome with 54 in the rituximab cohort and 29 in the NSIST cohort. The mean MGC score at the time of event or censor was 4.73 (SD 5.34) in the NSIST cohort and 5.69 (SD 5.61) in the rituximab cohort (t-test *p* = 0.18).

**Table 4:** Cox proportional hazards analysis for achievement of secondary composite treatment outcome (daily steroid dosage of ≤ 5 mg prednisolone equivalent and no rescue therapy with IVIG or PLEX within 4 weeks) of rituximab compared to second NSIST.

Group	Patients	Effective sample size (IPTW)	Events	b*	HR	95% CI	*SE*	Robust SE	*z*	*p*
NSIST	52	39.9	29		1.00 (ref)					
Rituximab	117	94.8	54	0.47	1.59	[0.95, 2.67]	0.16	0.26	1.77	0.077

In the sensitivity analyses, IPTW balance was achieved on all of the alternative cohorts, and while rituximab demonstrated an increased HR of achieving the composite outcome in all alternative models over the NSIST-treated population, none reached statistical significance, consistent with the primary analysis.

## Discussion

Treatment with rituximab in AChR-antibody positive patients did not demonstrate a statistically significant advantage compared to treatment with a second NSIST in analyses looking at time to achieving a composite clinical outcome (MGC ≤ 3, daily corticosteroid dose ≤ 5 mg prednisolone equivalent and no rescue therapies administered within the previous month). The wide confidence intervals observed preclude further inference with respect to the true effect, which could plausibly range between being less effective, broadly similar, to more effective than a second NSIST. We adjusted for the major cohort differences of worse baseline MGC score in the rituximab cohort (by IPTW) as well as the higher IST exposure in the rituximab cohort (by both IPTW and selection of NSIST patients on treatment with their second agent).

While analysis of the secondary outcome did not demonstrate a statistically significant hazard ratio difference between rituximab and a second NSIST, the hazard ratio was higher compared to the primary outcome. The secondary outcome focused on the steroid-sparing effects of these therapies without accounting for symptom severity as represented by the MGC score. This may suggest that the current MGBase cohort is underpowered to evaluate this effect properly. Despite not accounting for symptom severity in the outcome, the MGC score at the time of event or censor were still similar between the two cohorts, which suggests a degree of collinearity between corticosteroid usage and clinical severity.

Overall, the utility of rituximab in AChR MG remains equivocal, with some support from case-series and meta-analysis evidence. The landscape is further complicated by two recent, randomized controlled trials. The BeatMG study was designed as a futility analysis that randomized 52 AChR MG patients to standard therapy (which included other immunosuppressants) plus rituximab or placebo (15). It did not demonstrate increased odds that rituximab would achieve the primary outcome of steroid-sparing without worsening MG symptoms compared to placebo at 52 weeks. However, there was a suggestion that the rituximab cohort in BeatMG experienced fewer exacerbations (3 vs. 11) but remained underpowered to be statistically significant. The RINOMAX trial randomized 47 patients with new diagnoses of MG (BeatMG had no limitation on duration) to treatment with a single infusion of rituximab versus placebo (14). It demonstrated at 16 weeks that a significantly greater proportion of rituximab-treated patients compared to placebo achieved the primary composite outcome (71% vs. 29%) of minimal disease manifestation (QMG score ≤ 4) and a daily dose of prednisolone less than 10 mg. The authors propose that treatment with rituximab early in the disease course might circumvent the establishment of longer-lived plasma cells that no longer express CD20 (24), where greater efficacy might be seen with early use of rituximab rather than its current role as a second-line agent in later, more refractory disease. This could be explored explicitly in future analyses of MGBase, but currently, the cohort of patients who have received early treatment with rituximab prior to any NSIST exposure is small, consistent with general clinical practice.

Of note, rituximab has an established role in MuSK MG as demonstrated in previous systematic review (25). MuSK patients were excluded from this analysis, and further sensitivity analysis including seronegative patients did not significantly alter the outcome of the time-to-event analysis.

The study cohort that entered into the time-to-event analysis is largely representative of the greater MGBase cohort, except for thymectomy status, where a significantly greater proportion of patients in the study cohort had received thymectomies driven predominantly by patients who had thymoma. While a statistically significant difference in the worst MGC score was seen between the two cohorts, the absolute value was small.

Furthermore, as the MGBase registry matures and patient numbers increase, future analyses should look to stratify both the NSIST cohort as well the rituximab cohort. Resolving specific NSIST choices will allow comparison of rituximab directly against mechanistically distinct agents, and resolving rituximab usage patterns to more detailed granularity will provide highly relevant clinical guidance, which unfortunately is precluded in this analysis due to limited sample size.

### Limitations

Several limitations exist in this study. While a time-to-event analysis has been undertaken to maximize the power from the patient years captured in the registry, missing events and follow-up remains a source of bias. MGBase is a young database, and larger numbers with longer follow-up as the database develops may provide additional information. Usual limitations of registry studies with real-world data apply, including inconsistent or incomplete data capture and variability in clinical practice that may affect generalizability. Specific to a time-to-event analysis, measurements were taken from routine clinic visits to identify both baseline attributes and events, where visit density can be variable, potentially introducing a loss of sensitivity due to reporting delays in a time-to-event analysis. While a strict outcome measure was developed to achieve a meaningful clinical event, the attrition seen in registry patient numbers who did not have such a complete dataset risks introducing selection bias, despite comparable baseline characteristics between the study cohort and the overall MGBase cohort from which they were selected. Notably, a significant attrition of the cohort occurred when selecting patients treated with a second, different NSIST. This reflects clinical practice where rituximab is often used as a second-line treatment instead, thus accounting for the asymmetrical population size between the rituximab and NSIST-treated cohorts. Furthermore, the different clinical usage patterns between rituximab and NSISTs may introduce bias, where rituximab is generally used for a briefer duration than standard NSISTs reflecting clinical equipoise. While Schoenfeld diagnostics held validity, longer follow-up durations, given the short median rituximab treatment duration, could threaten the hazards assumption, as not enough events are yet available to power a time-varying analysis. While relevant and captured covariates were included in the IPTW covariates, unmeasured factors cannot be completely ruled out. In particular, while baseline MGC was balanced by IPTW, this score represents a single cross-sectional measurement of disease severity and likely under-captures underlying disease trajectory. In clinical practice, a rapidly deteriorating patient is more likely to receive higher-intensity treatment like rituximab, whereas slower-acting NSISTs may be reserved for more stable patients. This unmeasured confounding by indication – specifically the velocity of clinical worsening not captured by snapshot MGC score – cannot be fully resolved by adjusting for baseline MGC alone. The bias from this is likely to be against rituximab.

## Conclusion

In this observational study of AChR Ab positive MG patients, where a time-to-event analysis was undertaken on IPTW-balanced pseudo-cohorts to allow for causal inference, no statistically significant difference was detected between rituximab and a second NSIST in achieving a good clinical outcome as defined by a patient acceptable symptom state (MGC score ≤ 3), corticosteroid sparing (≤ 5 mg daily prednisolone dosage or equivalent) and reduced usage of rescue therapies (no use of IVIG or PLEX within the preceding month). It is noteworthy that our study focused on later stages of disease, where rituximab was used in those with refractory MG. Rituximab remains a biologically plausible and clinically relevant treatment option in MG, particularly given its targeted B-cell depletion mechanism and supportive evidence in other MG subtypes and clinical settings. Our findings should not preclude its consideration in individualised treatment strategies, especially in the context of early intervention or specific patient phenotypes, where its potential benefit warrants further investigation in prospective controlled studies.

## Data Availability

The raw data supporting the conclusions of this article will be made available by the authors, without undue reservation.
